# Supplementary material for: Unravelling reference bias in ancient DNA datasets
Source: Bioinformatics. 2024 Jul 3;40(7):btae436. doi: 10.1093/bioinformatics/btae436 (PMC11254355; doi:10.1093/bioinformatics/btae436)
Supplement: btae436_Supplementary_Data [file btae436_supplementary_data.zip › S1-S4_data_plots.zip!/S1-S4_data/rhino_sim_plots.zip!/rhino_sim_plots/bwaaln_rhino_div2_1end_by_MQ.pdf]

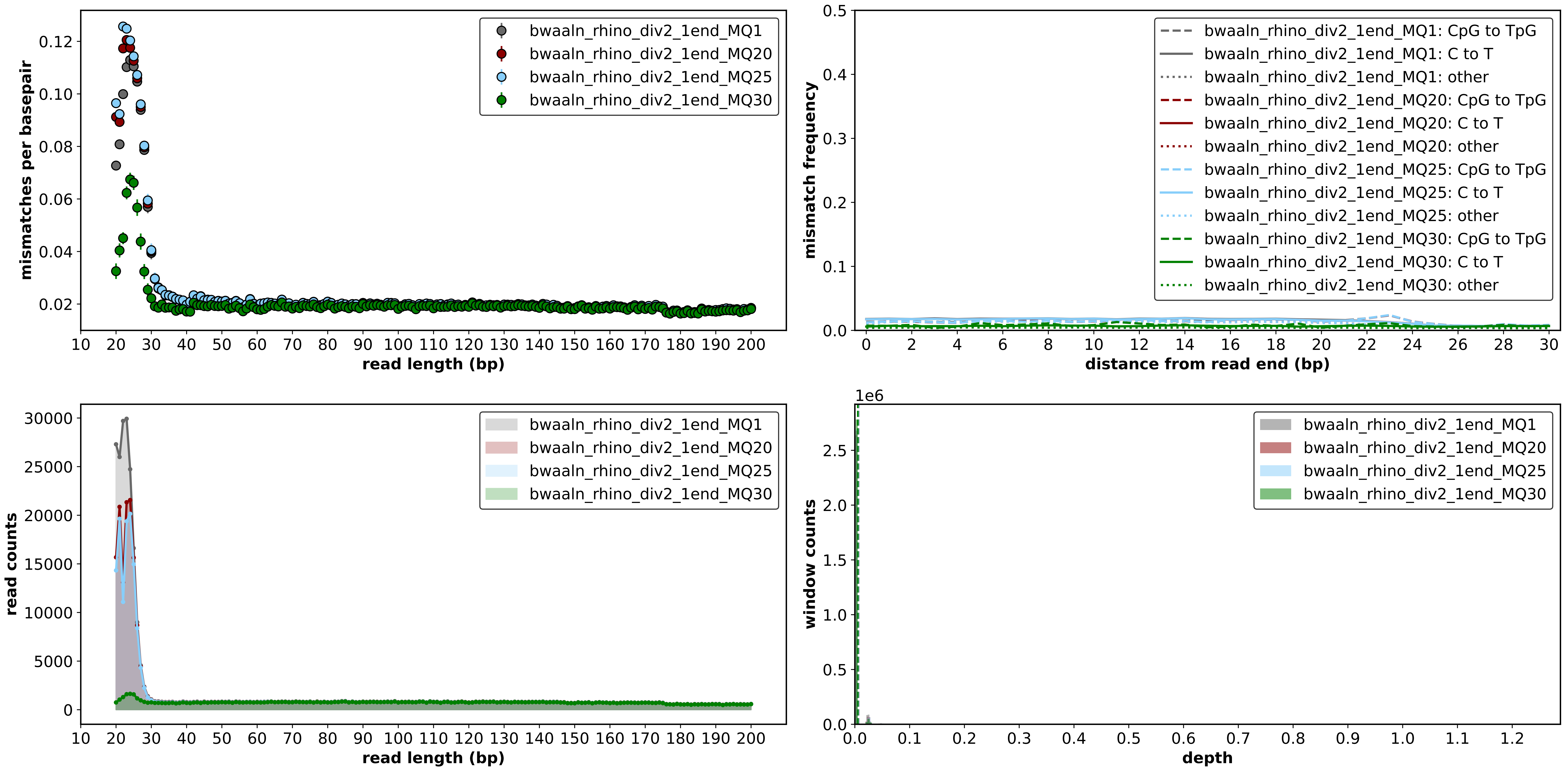
mismatches per basepair
0.12
0.10
0.08
0.06
0.04
0.02
10 20 30 40 50 60 70 80 90 100 110 120 130 140 150 160 170 180 190 200
read length (bp)
bwaaln_rhino_div2_1end_MQ1
bwaaln_rhino_div2_1end_MQ20
bwaaln_rhino_div2_1end_MQ25
bwaaln_rhino_div2_1end_MQ30
mismatch frequency
0.5
0.4
0.3
0.2
0.1
0.0
0 2 4 6 8 10 12 14 16 18 20 22 24 26 28 30
distance from read end (bp)
bwaaln_rhino_div2_1end_MQ1: CpG to TpG
bwaaln_rhino_div2_1end_MQ1: C to T
bwaaln_rhino_div2_1end_MQ1: other
bwaaln_rhino_div2_1end_MQ20: CpG to TpG
bwaaln_rhino_div2_1end_MQ20: C to T
bwaaln_rhino_div2_1end_MQ20: other
bwaaln_rhino_div2_1end_MQ25: CpG to TpG
bwaaln_rhino_div2_1end_MQ25: C to T
bwaaln_rhino_div2_1end_MQ25: other
bwaaln_rhino_div2_1end_MQ30: CpG to TpG
bwaaln_rhino_div2_1end_MQ30: C to T
bwaaln_rhino_div2_1end_MQ30: other
read counts
30000
25000
20000
15000
10000
5000
0
10 20 30 40 50 60 70 80 90 100 110 120 130 140 150 160 170 180 190 200
read length (bp)
bwaaln_rhino_div2_1end_MQ1
bwaaln_rhino_div2_1end_MQ20
bwaaln_rhino_div2_1end_MQ25
bwaaln_rhino_div2_1end_MQ30
1e6
window counts
2.5
2.0
1.5
1.0
0.5
0.0
0.0 0.1 0.2 0.3 0.4 0.5 0.6 0.7 0.8 0.9 1.0 1.1 1.2
depth
bwaaln_rhino_div2_1end_MQ1
bwaaln_rhino_div2_1end_MQ20
bwaaln_rhino_div2_1end_MQ25
bwaaln_rhino_div2_1end_MQ30
